# Anxiolytic and Antiepileptic Properties of the Aqueous Extract of *Cissus quadrangularis* (Vitaceae) in Mice Pilocarpine Model of Epilepsy

**DOI:** 10.3389/fphar.2018.00751

**Published:** 2018-07-17

**Authors:** Fleur C. O. Moto, Aren Arsa’a, Gwladys T. Ngoupaye, Germain S. Taiwe, Jacqueline S. K. Njapdounke, Antoine K. Kandeda, Gisele C. N. Nkantchoua, Jean P. Omam Omam, Simon Pale, Nadege E. Kouemou, Espoir R. Ayissi Mbomo, David B. Pahaye, Lucie Ojong, Veronique Mairara, Elisabeth Ngo Bum

**Affiliations:** ^1^Department of Biological Sciences, Higher Teachers’ Training College, University of Yaoundé I, Yaoundé, Cameroon; ^2^Department of Biological Sciences, Faculty of Science, University of Ngaoundéré, Ngaoundéré, Cameroon; ^3^Department of Animal Biology, Faculty of Science, University of Dschang, Dschang, Cameroon; ^4^Department of Zoology and Animal Physiology, Faculty of Sciences, University of Buea, Buea, Cameroon; ^5^Department of Animal Biology and Physiology, Faculty of Science, University of Yaoundé I, Yaoundé, Cameroon; ^6^Center of Medical Research, Institute of Medical Research and Studies on Medicinal Plants, Yaoundé, Cameroon; ^7^Department of Fundamental Sciences, Faculty of Mines and Petroleum Industries, University of Maroua, Maroua, Cameroon

**Keywords:** *C. quadrangularis*, anticonvulsant, anxiolytic, pilocarpine, status epilepticus, epileptogenesis

## Abstract

*Cissus quadrangularis* (*C. quadrangularis*) is a plant of the Vitaceae family known for its anticonvulsant effects in traditional medicine. The objective of this study was to elucidate the anxiolytic and antiepileptic effects of aqueous extract of *C. quadrangularis*. The mice were divided into different groups and treated for seven consecutive days as follows: a negative control group that received distilled water, po, four test groups that received four doses of the plant (37.22, 93.05, 186.11, and 372.21 mg/kg, po), and a positive control group that received sodium valproate (300 mg/kg, ip). One hour after the first treatment (first day), epilepsy was induced by intraperitoneal administration of a single dose of pilocarpine (360 mg/kg). On the seventh day, the anxiolytic effects of the extract were evaluated in the epileptic mice using the elevated plus maze (EPM) and open field (OP) paradigms. Antioxidant activities and the involvement of gabaergic neurotransmission were determined by measuring the levels of malondialdehyde, reduced glutathione (GSH), GABA, and GABA-transaminase (GABA-T) in the hippocampus of sacrificed epileptic mice. The results show that the extract of *C. quadrangularis* significantly and dose-dependently increased the latency to clonic and generalized tonic–clonic seizures and decreased the number and duration of seizures. In the EPM, the extract of *C. quadrangularis* significantly increased the number of entries and the time spent into the open arms and reduced the number of entries and the time spent into the closed arms as well as the number of rearing. The extract of *C. quadrangularis* also increased the number of crossing, and the time spent in the center of the OP. The level of MDA and the activity of GABA-T were significantly decreased by the extract of *C. quadrangularis* while reduced GSH and GABA levels were increased. The results suggest that the anticonvulsant activities of *C. quadrangularis* are accompanied by its anxiolytics effects. These effects may be supported by its antioxidant properties and mediated at least in part by the GABA neurotransmission.

## Introduction

Status epilepticus (SE) is the first manifestation of epilepsy in approximately 50% of patients (Chapman et al., 2001). Generalized tonic–clonic SE is the most dangerous form (Chen and Wasterlain, 2006). It is characterized by continuous seizures or the succession of seizures without the improvement of consciousness over a period of 30 min (Gastaut, 1973; Proceedings of the American Epilepsy Society Course, 1993; Dupont and Crespel, 2009). Normal cerebral activity is based on both excitatory and inhibitory actions. But in the epileptic focus of the pilocarpine SE models, which are pharmaco-resistant models, 20% of the neurons would be excited by GABA (Cohen et al., 2002). The excitatory role of GABA is due to molecular alterations that affect the natural function of GABA in epileptic tissue (Huberfeld et al., 2007). It characterizes the silent phase: epileptogenesis groups together a series of events during which irreversible neuronal lesions appear. The excitation (glutamate + GABA) becomes much too strong and the inhibition much too weak and leads to the establishment of a chronic state characterized by the occurrence of abnormal discharges in the neurons (Cherubini et al., 2011) hence the recurrence of spontaneous crises (Cavalheiro et al., 2006). These pathophysiological changes lead to the development of comorbidities related to epilepsy. (Faure, 2016). It has been shown that during epileptogenesis, the occurrence of anxiety disorders may be the consequence of lesions located especially in the amygdala and other structures playing a role in emotions (Faure, 2016) in the same way reactive oxygen species (ROS) overproduction (Azam et al., 2010). The overproduction of ROS causes the oxidation of the polyunsaturated fatty acids at the origin of the formation of hydroperoxides and aldehydes. Their biological activities cause the alteration of the structure of cell membranes and the disruption of these membranes (Sies and Stahl, 1995; Bonnefont-Rousselot et al., 2003), hence the cellular apoptosis (Dröge, 2002). Oxidative stress can be assessed by the dosage of MDA, compound resulting from the peroxidation of lipids, by the measurement of non-enzymatic systems such as GSH. GSH is a cofactor of the ROS detoxification pathways formed, the regulation of the redox potential, and the reduction of oxidation of the thiols groups of the proteins (Sohal and Weindruch, 1996; Beckman and Ames, 1998).

The goal of the treatment of the SE is to obtain the fast and lasting stop of the crises. None of the antiepileptic drugs available today have the properties of the ideal drug in terms of efficacy and tolerance (Ichai et al., 1996). These limits have developed more interest for the use of medical plants. In Africa, herbal medicine still plays an important role in disease management, mainly in very low-income populations (Geoffrey and Kirby, 1996). In addition, certain diseases such as epilepsy, depression, and anxiety in Cameroon are considered as mystical diseases (personal communications). *Cissus quadrangularis* Linn (Vitaceae) (*C. quadrangularis*) is a medicinal plant used in traditional medicine in northern Cameroon for the treatment of epilepsy (Ngo Bum et al., 2008). *C. quadrangularis* is native to India and Malaysia and grows in savannah areas in Africa (Cameroon, Mali, Mauritania, Senegal, Somalia, and Chad; Dumas-Champion, 1997; Arbonier, 2000). In traditional medicine, the plant is used to treat epilepsy (Ngo Bum et al., 2008; Shah, 2011), convulsion (Shah, 2011), hemorrhoids, anorexia, indigestion, and asthma (Rajpal, 2002). Chemical studies have shown the presence of sterols, steroids, tannins, flavonoids (quercetin and kaempferols), carotenes, ascorbic acid, and linoleic acid in *C. quadrangularis* (Sen, 1966; Saburi et al., 1999; Murthy et al., 2003). Pharmacological studies on fresh leaves and roots have shown that *C. quadrangularis* has antioxidant, antibacterial, analgesic, and neurosedative (Amos et al., 2001; Murthy et al., 2003; Viswanatha et al., 2006). The studies on the stems have revealed anticonvulsant and sedative properties (Ngo Bum et al., 2008). A three-month subchronic toxicity study suggested that *C. quadrangularis* administered orally at a dose of 3 g/kg is non-toxic (Attawish et al., 2002). The purpose of this study was to investigate the antiepileptogenic and anxiolytic properties of *C. quadrangularis* following the study of Ngo Bum et al. (2008).

## Materials and Methods

### Plant

The entire plant was harvested early in the morning in the Mogode village, Far-North Region, Cameroon, in June. A voucher specimen has been deposited at the Yaounde national herbarium on the number 36966 HNC/Cam.

### Preparation of the Aqueous Extract of *C. quadrangularis* (AECQ)

The fresh and whole plant of *C. quadrangularis* was cut into pieces and air dried at room temperature. The powder was added to distilled water (10 g in 140 ml) and boiled for 20 min. Following cooling at room temperature, the solution obtained was filtered with Whatman No1 filter paper. The filtrate was considered as the stock solution. The amount of dry matter in the extract was determined by evaporating water in a drying oven (50°C). A solid residue (2.17 g) was obtained. The yield of extraction was 21.7%, and the stock solution dose was 372.21 mg/kg. The other doses used in the study (186.1, 93.05, and 37.22 mg/kg) were obtained by dissolving the stock solution in distilled water at ratios of 1/2, 1/4, and 1/10, respectively. The procedure for obtaining the dose used by traditional healers has been respected.

### Animals and Experimental Design

White *Mus musculus* swiss mice of both sexes, weighting 18–28 g were used for this study. These mice came from the National Veterinary Laboratory (LANAVET, Garoua, Cameroon), they were raised under standard conditions at the University of Ngaoundere (Ngaoundere, Cameroon), and fed with water and food at will. Animals were maintained on a 12 h/12 h light/dark cycle. Animals were acclimated to laboratory conditions 72 h before starting the experiments. The study was carried out in accordance with the Cameroon National Ethical Committee (Ref No. FW-IRB00001954, 22 October 1987). The authorization number (1244 CEI-UDO/12/2017/A) was given and the study was done also in conformation with the international regulation, minimizing the number of mice used and their suffering.

The mice were organized into nine lots of six mice each. The first day of the test, seven lots of six mice received the following treatments:

• lot 1, normal control group (DW + DW) receiving only distilled water (10 ml/kg, p.o.);
• lot 2, negative control group (DW + Pilo) receiving distilled water (10 ml/kg, p.o.);
• lots 3–6, test lots receiving the different doses of EACQ (37.22, 93.05, 186.11, and 372.21 mg/kg, p.o.);
• lot 7, positive control group (VS) receiving sodium valproate (300 mg/kg, i.p., Sigma-Aldrich).

The females used were non-pregnant since male mice were isolated from females before and during experimental testing. Males and females were distributed equally in the different lots.

The procedure for induction of SE by pilocarpine is based on previous studies by Turski et al. (1983), Curia et al. (2008), and Magnin (2014). Forty minutes after the first treatment, to reduce the peripheral effects of pilocarpine, each lot received *N*-methyl-scopolamine (1 mg/kg, i.p., Sigma-Aldrich), except lot 1. Twenty minutes after administration of *N*-methyl-scopolamine, lot 1 still received distilled water; all the other lots were treated with a single dose of pilocarpine hydrochloride (360 mg/kg, i.p., Sigma-Aldrich). Each mouse was returned to its initial cage and observed individually for 6 h to determine the severity and duration of acute epileptic seizures compared to the Racine scale (Racine, 1972). After about 20 min, some animals became hypoactive and then displayed successively oro-facial movements, salivation, eye blinking, twitching of vibrissae, yawning, and generalized convulsions. Only mice that developed stage 5 attacks of the Racine scale (tonic–clonic seizures with loss of righting reflex) for two consecutive periods were retained for this study.

### Study of Anticonvulsant Effects of *C. quadrangularis* Extract During Epileptogenesis

Twenty three hours after the injection of pilocarpine, the mice received once again their different treatments (mice were treated with distilled water for lots 1 and 2, the respective doses of the plant for lots 3–6, and sodium valproate for lot 7). After 1 h, the behavioral parameters were evaluated for each mouse for a period of 30 min. The parameters observed were the latency time to the first tonic–clonic seizures, the duration of tonic–clonic seizures, and the number of tonic–clonic seizures. From the third to the seventh day, the mice were treated as on the second day.

### Behavioral Assessment

Seven days the administration of pilocarpine and 1 h after the administration of the different daily treatments, the mice were observed for 5 min in the elevated plus maze (EPM; Pellow et al., 1985; Rodgers and And Johnson, 1995) and after in the open field (OP) paradigm (Lister, 1990).

#### Elevated Plus Maze Test

The test in the EPM has taken place in a quiet room. The method used in this study was that described by Rodgers and Dalvi (1997). It consisted of placing a mouse in the center of the EPM with two open arms and two closed arms. The mice were free to explore the maze during 5 min (Anderson et al., 2000). A group of mice receiving diazepam (3 mg/kg) was added to this test. The number of entries and the time spent in the center and in the different arms were recorded. The number of rearing and head dipping were recorded too. After observing the behavior of a mouse, the labyrinth was cleaned with alcohol.

#### Open Field Test

The method used in our experiment was that described by Belzung (1999). The OP test is commonly used to evaluate the level of locomotor activity, exploration, and emotional reactivity in rodents (Blizard, 1989; Van der Staay et al., 1990; Bronikowski et al., 2001). A group of mice receiving diazepam (0.3 mg/kg) was added to this test. The mice were placed one after the other in the center of the experimental device. The behavior of each mouse was observed for a period of 5 min. Several behavioral parameters were evaluated: the number of crossing (number of lines crossed), the number of grooming (when the animal cleans its body), the frequency of rearing (when the animal stands up on its hind legs and bears on the edges of the experimental device), the time spent in the center of the experimental device, and the fecal boli produced.

### Biochemical Tests

At the exit of the OP, the animals were immediately sacrificed. Their brain was dissected and cleaned with ice-cold saline solution (0.9%, w/v) to remove the hippocampus. Then, the hippocampi were weighed. To perform biochemical analyzes, 10% (w/v) homogenates prepared with ice-cold 0.1 M phosphate buffer (pH 7.4) were centrifuged (10,000 ×*g*, 15 min). The aliquots of the supernatants were collected and used to measure, according to different protocols, the level of reduced glutathione (GSH), malondiadéhyde (MDA), GABA, and GABA-transaminase (GABA-T).

#### GSH Level

Glutathione was measured using the method of Ellman (1959). Briefly, 1500 μl of DNTB and 500 μl of Tris-HCl (Sigma-Aldrich) buffer (50 mM, pH 7.4) were added to a blank tube containing 100 μl of Tris-HCl buffer (50 mM, pH 7.4) or to test tubes containing tissue homogenates (100 μl). The mixture solution was incubated for 1 h, and the absorbance was read against the blank at 412 nm. The GSH concentration was calculated using an extinction coefficient of 13,600 mol^−1^cm^−1^. The concentration of GSH was expressed as μmol/g of protein in the tissue.

#### MDA Level

The method of Wilbur et al. (1949) for MDA determination was used. Briefly, distilled water (250 μl) and homogenate (20 μl) were introduced in the control tube and in the test tubes, respectively. Then, 250 μl of Tris-HCl buffer (50 mM, pH 7.4), 500 μl of trichloroacetic acid (20%, Sigma-Aldrich), and 1000 μl of thiobarbituric acid (0.67%, Sigma-Aldrich) were added. The mixture solution was heated in a water-bath (90°C, 10 min). After cooling at room temperature, the tubes were centrifuged (3000 rpm, 15 min). The absorbance of the pink-colored supernatant was measured against the blank at 530 nm. The MDA concentration was calculated using an extinction coefficient of 1.56 × 105 mmol^−1^cm^−1^. MDA level was expressed in μmol/g of protein in the tissue.

#### GABA Level

The level of hippocampal GABA was determined by the colorimetric assay of mouse brain homogenates as described by Lowe et al. (1958). The working reagent consisted of a mixture of 0.2 ml of 0.14 M ninhydrin solution prepared in a solution of carbonate–bicarbonate buffer (0.5 M, pH 9.9), and 0.1 ml glacial trichloroacetic acid (10%, Sigma-Aldrich). A homogenate sample of 100 μl was taken and introduced into the working reagent; the mixture was incubated at 60°C in a water bath for 30 min. After cooling, the mixture was introduced into a solution of copper tartrate (5 ml, Sigma-Aldrich). The mixture was kept at a temperature of 25°C for 10 min. The fluorescence resulting from the reaction between ninhydrin and GABA in the basic medium was measured using a spectrofluorometer (377/451 nm). The measured absorbance was proportional to the concentration of GABA in the homogenates. A standard GABA solution was prepared in parallel from different masses of GABA (100, 150, 200, 250, 300, 350, and 400 μg) each mixed with 1.5 mg of glutamate dissolved in 0.1 ml of ice-cold solution TCA (10% Sigma-Aldrich). The concentration of GABA in the hippocampal homogenate samples was determined by the measurement of the formed fluorescent product resulting from the reaction of GABA with ninhydrin an alkaline medium, in the presence of glutamate (Sutton and Simmonds, 1974). The GABA content in brain was expressed in mg/g of brain tissue.

#### GABA-T Activity

The activity of GABA-T was evaluated by the colorimetric assay method of Nayak and Chatterjee (2003). Graduated 10 ml tubes were used for this assay; 15 μmol of I-oxoglutarate, 15 μmol of GABA, 10 μg of pyridoxal phosphate, 0.1 ml of homogenate supernatant (test tubes), and 0.1 ml of methanol 5% were introduced into these tubes (white tube). The final volume of the mixture was made up to 3 ml with Tris-HCl buffer (50 mM, pH 7.4). These tubes were then incubated at 37°C for 30 min in a 96-well microplate. The reaction was finalized by adding 0.5 ml of glacial TCA (20%, Sigma-Aldrich). The semialdehyde succinic acid produced during the incubation of the mixture was estimated spectrophotometrically and the absorbance was read at 610 nm after 30 and 90 s against the blank. Staining of the semialdehyde succinic acid complex and 3-methyl-2-benzothiazole-2-hydrazone in the presence of 12% FeCl_3_ was measured and was proportional to the concentration of GABA-T in the homogenates. The activity of GABA-T was estimated in pg/min/mg of tissue.

### Statistical Analysis

Statistical analysis of the values obtained and the construction of the graphs was performed using XL Stat software: GraphPad Prism version 5.03, Microsoft Office Excel 2013 version 15.0.4420.11017. The results were expressed as mean ± standard error of means (SEM) or as percentages. The different values were compared using one-way analysis of variance (ANOVA) and when differences existed, the Tukey (HSD) multiple comparison tests were used as the *post hoc* test. *P* < 0.05 was considered significant.

## Results

### Effects of *C. quadrangularis* on Seizures Induced by Pilocarpine

#### Latency to Status Epilepticus

**FIGURE 1:**
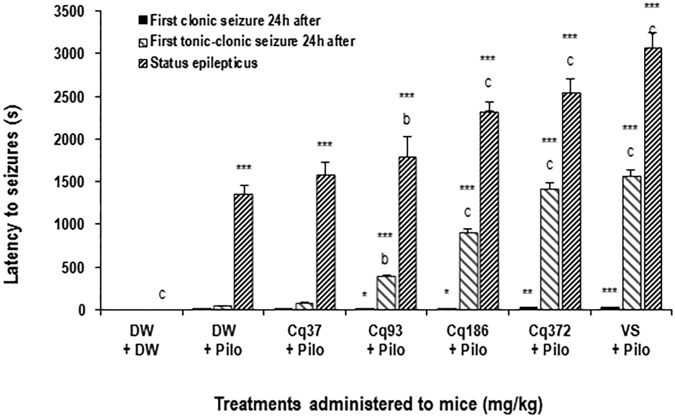
Effects of *C. quadrangularis* extract on the latency to status epilepticus, to first clonic and tonic–clonic seizures 24 h after status epilepticus. Results are expressed as mean ± SEM for six animals. Data were analyzed by one-way ANOVA, followed by Tukey (HSD). ^∗∗∗^*p* < 0.001, vs control animals (DW + DW group) receiving only distilled water; ^b^*p* < 0.01 and ^c^*p* < 0.001 vs disease control animals (DW + Pilo group) receiving distilled water and pilocarpine (360 mg/kg). DW, distilled water; Cq, *Cissus* *quadrangularis*; Pilo, pilocarpine; SV, sodium valproate (300 mg/kg). ^∗^*p* < 0.05, ^∗∗^*p* < 0.01.

The latency time increased significantly and dose-dependently by 31.76% (*p* < 0.01), 71.37, and 87.92% (*p* < 0.001) in the mice treated with EACQ 93; 186 and 372 mg/kg, respectively, compared to the DW + Pilo group. Latency increased to 126.41% (*p* < 0.001) with sodium valproate relative to the DW + Pilo group (**Figure 1**).

#### Latency to First Clonic and Tonic–Clonic Seizures 24 h After Status Epilepticus

It appears that the time of onset of the first clonic and tonic–clonic seizures in mice in the DW + Pilo groups (4 and 43 s, respectively) are not significant compared to the DW + DW group. The onset of seizure time increased significantly and dose-dependently in mice treated with EACQ. The latency times of the first tonic–clonic seizures were 393, 904, and 1422 (*p* < 0.001) at the doses 93, 186, and 372 mg/kg. Sodium valproate increased the latency to 1567 s (*p* < 0.001; **Figure 1**).

#### Clonic Seizures Number and Duration

**FIGURE 2:**
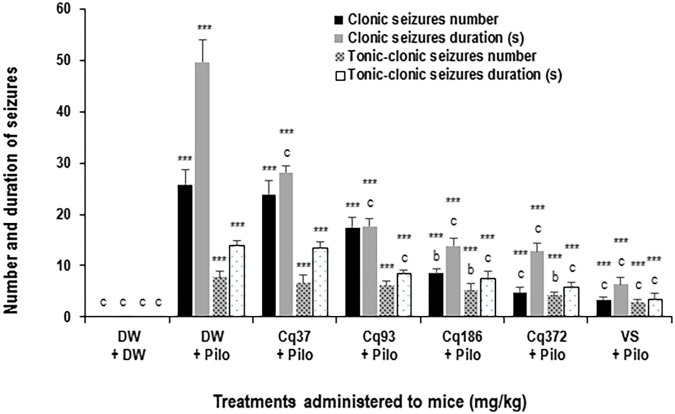
Effects of *C. quadrangularis* extract on the number and duration of clonic and tonic–clonic seizures. Results are expressed as mean ± SEM for six animals. Data were analyzed by one-way ANOVA, followed by Tukey (HSD). ^∗∗∗^*p* < 0.001, vs control animals (DW + DW group) receiving only distilled water; ^b^*p* < 0.01 and ^c^*p* < 0.001 vs disease control animals (DW + Pilo group) receiving distilled water and pilocarpine (360 mg/kg). DW, distilled water; Cq, *Cissus* *quadrangularis*; Pilo, pilocarpine; SV, sodium valproate (300 mg/kg).

**FIGURE 3:**
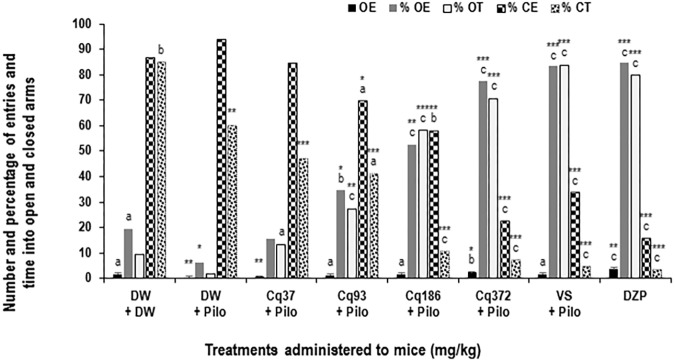
Effects of *C. quadrangularis* extract on the number of open arm entries, on the percentages of entries and time in opened and closed arms. Results are expressed as mean ± SEM and as percentage for six animals. Data were analyzed by one-way ANOVA, followed by Tukey (HSD). ^∗^*p* < 0.05, ^∗∗^*p* < 0.01, and ^∗∗∗^*p* < 0.001, vs control animals (DW + DW group) receiving only distilled water; ^a^*p* < 0.05, ^b^*p* < 0.01, and ^c^*p* < 0.001 vs disease control animals (DW + Pilo group) receiving distilled water and pilocarpine (360 mg/kg). DW, distilled water; Cq, *Cissus* *quadrangularis*; Pilo, pilocarpine; SV, sodium valproate (300 mg/kg); DZP, diazepam (3 mg/kg).

*Cissus quadrangularis* decreased the number and duration of generalized clonic seizures from 25.8 ± 2.7 in the DW + Pilo group to 8.5 ± 0.8 (*p* < 0.01) and 4.8 ± 0.9 (*p* < 0.001) in the groups of mice administered with 186 and 372 mg/kg of *C.* *quadrangularis*, respectively (**Figure 3**). The number of seizure continued to decrease to 3.3 ± 0.5 (*p* < 0.001) in the group of mice administered with sodium valproate (**Figure 2**). The duration of generalized clonic seizures was reduced from 49.6 ± 4.4 s in the DW + Pilo group to 13.8 ± 1.6 and 12.8 ± 1.4 s (*p* < 0.001) in the groups of mice administered with *C. quadrangularis* at doses 186 and 372 mg/kg, respectively (**Figure 3**). Sodium valproate reduced the generalized clonic seizures to 6.3 ± 1.5 s (*p* < 0.001) compared to the DW + Pilo group of mice.

#### Tonic–Clonic Seizures Number and Duration

*Cissus quadrangularis* decreased the number and duration of generalized tonic–clonic seizures from 7.6 ± 1.2 in the DW + Pilo group to 4.1 ± 0.7 (*p* < 0.01) in the group of mice administered with 372 mg/kg of *C.* *quadrangularis* (**Figure 3**). The number of seizure continued to decrease to 2.6 ± 0.8 (*p* < 0.001) in the group of mice administered with sodium valproate (**Figure 3**). The duration of generalized tonic–clonic seizures was reduced from 13.8 ± 0.9 s in the DW + Pilo group to 7.3 ± 1.5 s (*p* < 0.001) and 5.6 ± 1.2 s (*p* < 0.001) in the groups of mice administered with *C. quadrangularis* at doses 186 and 372 mg/kg, respectively (**Figure 2**). Sodium valproate reduced the generalized tonic–clonic seizures by 75.92% (*p* < 0.001) compared to the DW + Pilo group of mice.

### Anxiolytic-Like Effects of *C. quadrangularis* on the EPM Test

Diazepam and *C. quadrangularis* at 372 mg/kg dose significantly increased the number of open-arm entries from 0.66 ± 0.44 in the DW + Pilo group to 3.66 ± 0.44 (*p* < 0.001) and 2.33 ± 0.44 (*p* < 0.001), respectively (**Figure 3**). The percentages of entries and time spent in the open arms increased from 10.25 and 4.27 in the DW + Pilo group up to 83.33, 83.33 for the group SV (*p* < 0.001) and 84.61, 79.94 for the DZP group (*p* < 0.001; **Figure 3**). Mice treated with *C. quadrangularis* at doses 93, 186, and 372 mg/kg increased also significantly and dose dependently the percentages of entries and time spent in the open arms (*p* < 0.001; **Figure 3**). Like diazepam, *C. quadrangularis* reduced the number of closed arm entries (**Table 1**) and the percentage of entries and time in closed arms (*p* < 0.001; **Figure 3**). In addition, the numbers of rears and dips were reduced by both diazepam and *C. quadrangularis* (**Table 1**).

**Table 1:** Effect of *C. quadrangularis* in EPM test: closed arm entries, total arms entries, ratio OE/TE vs CE/TE, rearing, and head dipping.

Treatments	Doses (mg/kg)	Closed entries	Total entries	Ratio	Rearing	Head dipping
DW + DW	− + −	7.50 ± 0.83	8.66 ± 1.11	22.22	8.66 ± 0.38	2.66 ± 0.88
DW + Pilo	− + 360	5.16 ± 0.83^∗^	5.50 ± 0.66^∗^	06.45	14.33 ± 1.23^∗∗∗^	5.83 ± 0.76^∗∗^
*Cq* + Pilo	37 + 360	3.66 ± 0.44^∗∗∗^	4.33 ± 0.66^a^∗∗∗	18.18^∗∗^	7.83 ± 0.88^c^	3.50 ± 1.16
*Cq* + Pilo	93 + 360	2.66 ± 0.66^c∗∗∗^	3.83 ± 0.27^b∗∗∗^	50.01^c∗^	5.83 ± 1.55^c^	2.66 ± 2.00^b^
*Cq* + Pilo	186 + 360	1.83 ± 0.61^c∗∗∗^	3.16 ± 0.88^c∗∗∗^	90.91^c∗∗∗^	3.50 ± 2.16^c∗∗∗^	2.33 ± 0.66^c^
*Cq* + Pilo	372 + 360	0.66 ± 0.44^c∗∗∗^	1.66 ± 0.44^c∗∗∗^	350.01^c∗∗∗^	1.16 ± 0.94^c∗∗∗^	1.16 ± 0.55^c^
SV + Pilo	300 + 360	0.66 ± 0.44^c∗∗∗^	2.00 ± 0.66^c∗∗∗^	250.01^c∗∗∗^	1.50 ± 0.66^c∗∗∗^	0.66 ± 0.66^c^
DZP + DW	3 + −	0.66 ± 0.44^c∗∗∗^	4.33 ± 0.66^a∗∗∗^	550.01^c∗∗∗^	1.16 ± 0.27^c∗∗∗^	0.50 ± 0.66^c^

*Results are expressed as mean ± SEM, N = 6. Data were analyzed by one-way ANOVA, followed by Tukey (HSD). ^∗^p < 0.05, ^∗∗^p < 0.01, and ^∗∗∗^p < 0.001, vs control animals (DW + DW group) receiving only distilled water; ^a^p < 0.05, ^b^p < 0.01, and ^c^p < 0.001 vs disease control animals (DW + Pilo group) receiving distilled water and pilocarpine (360 mg/kg). DW, distilled water; Cq, Cissus quadrangularis; Pilo, pilocarpine; SV, sodium valproate (300 mg/kg); DZP, diazepam (3 mg/kg).*

### Anxiolytic-Like Effects of *C. quadrangularis* on the OF Test

The OF test revealed that *C. quadrangularis* increased in a dose-dependent manner, the number of crossing and grooming from 13.16 ± 1.16 and 0.66 ± 0.44 in the DW + Pilo groups to 39.83 ± 2.5 and 1.83 ± 0.55, respectively, at the dose of 372 mg/kg. The increase was also observed in the time spent by mice in the center from 2.50 ± 0.66 s in the DW + Pilo group to 32.83 ± 3.72 s at the dose of 372 mg/kg (*p* < 0.001). At the same dose of 372 mg/kg, *C. quadrangularis* decreased the number of rears and the mass of fecal boli (*p* < 0.001) and (*p* < 0.001), respectively. The effect was the same with diazepam (0.3 mg/kg; **Table 2**).

**Table 2:** Effect of *C. quadrangularis* in open field test: rearing, crossing, grooming, center time, and quantity of fecal boli.

Treatments	Doses (mg/kg)	Rearing	Crossing	Grooming	Center time (s)	Fecal boli (g)
DW + DW	− + −	6.33 ± 0.66	6.33 ± 0.83	1.50 ± 0.50	7.16 ± 0.83	0.29 ± 0.10
DW + Pilo	− + 360	10.83 ± 2.16^∗∗∗^	13.16 ± 1.16	0.66 ± 0.44	2.50 ± 0.66	0.41 ± 0.32
*Cq* + Pilo	37 + 360	4.83 ± 2.16^c^	18.66 ± 2.11^∗∗∗^	1.33 ± 0.44	5.83 ± 0.83	0.08 ± 0.05^a^
*Cq* + Pilo	93 + 360	4.33 ± 1.33^c^	26.66 ± 2.11^c∗∗∗^	1.33 ± 0.55	11.83 ± 0.88^c^	0.03 ± 0.04^b^
*Cq* + Pilo	186 + 360	2.83 ± 0.61^c∗∗^	35.16 ± 2.55^c∗∗∗^	1.33 ± 0.44	23.16 ± 1.16^c∗∗∗^	0.03 ± 0.04^b^
*Cq* + Pilo	372 + 360	2.50 ± 0.50^c∗∗^	39.83 ± 2.50^c∗∗∗^	1.83 ± 0.55	32.83 ± 3.72^c∗∗∗^	0.01 ± 0.03^b^
SV + Pilo	300 + 360	1.83 ± 1.11^c∗∗∗^	40.83 ± 3.83^c∗∗∗^	2.33 ± 0.77^b^	35.16 ± 4.16^c∗∗∗^	0.06 ± 0.08^b^
DZP + DW	3 + −	1.50 ± 0.50^c∗∗∗^	49.33 ± 5.22^c∗∗∗^	2.16 ± 0.27^b^	39.16 ± 1.88^c∗∗∗^	0.02 ± 0.03^b^

*Results are expressed as mean ± SEM, N = 6. Data were analyzed by one-way ANOVA, followed by Tukey (HSD). ^∗∗^p < 0.01 and ^∗∗∗^p < 0.001, vs control animals (DW + DW group) receiving only distilled water; ^a^p < 0.05, ^b^p < 0.01, and ^c^p < 0.001 vs disease control animals (DW + Pilo group) receiving distilled water and pilocarpine (360 mg/kg). DW, distilled water; Cq, Cissus quadrangularis; Pilo, pilocarpine; SV, sodium valproate (300 mg/kg); DZP, diazepam (0.3 mg/kg).*

### Levels of GHS, MDA, GABA, and GABA-T

#### GSH Level

The GSH rate decreased significantly in the DW + Pilo group (113.83 ± 1.22 mol/g tissue) compared to the DW + DW group (202.33 ± 1.33 mol/g tissue; *p* < 0.001). *C. quadrangularis* induced a dose-dependent increase of the GSH level, from 113.83 ± 1.22 mol/g of tissue to 160.16 ± 8.16 (*p* < 0.001) and 186.16 ± 4.55 mol/g of tissue (*p* < 0.001) at the doses of 186 and 372 mg/kg, respectively. Sodium valproate and diazepam groups increased GSH level to 189.66 ± 4.88 and 211.16 ± 22.27 (*p* < 0.001), respectively, relative to the DW + Pilo group (**Table 3**).

**Table 3:** Effects of *C. quadrangularis* on oxidative stress markers in hippocampi of pilocarpine-injected mice.

Treatments	Doses (mg/kg)	GHS (μmol/g)	MDA (μmol/g)	GABA (μg/g)	GABA-T (pg/min/mg)
DW + DW	− + −	202.33 ± 1.33	129.83 ± 5.11	397.50 ± 1.66	47.16 ± 2.11
DW + Pilo	− + 360	113.83 ± 1.22^∗∗∗^	400.66 ± 4.44^∗∗∗^	257.16 ± 8.44^∗∗∗^	112.33 ± 4.88^∗∗∗^
*Cq* + Pilo	37 + 360	126.16 ± 5.55^∗∗∗^	385.16 ± 4.44^c∗∗∗^	277.16 ± 9.38^b ∗∗∗^	89.83 ± 5.44^a∗∗∗^
*Cq* + Pilo	93 + 360	139.83 ± 3.77^c∗∗∗^	259.33 ± 9.55^a∗∗∗^	310.16 ± 2.88^a∗∗∗^	80.83 ± 6.22^a∗∗∗^
*Cq* + Pilo	186 + 360	160.16 ± 8.16^a∗∗∗^	243.33 ± 4.44^a∗∗∗^	369.83 ± 10.11^a∗∗∗^	63.16 ± 2.16^a∗∗∗^
*Cq* + Pilo	372 + 360	186.16 ± 4.55^a^	141.16 ± 5.44^a^	393.66 ± 5.77^a^	45.83 ± 2.83^a^
SV + Pilo	300 + 360	189.66 ± 4.88^a^	130.66 ± 3.55^a^	391.66 ± 3.33^a^	52.66 ± 5.11^a^
DZP + DW	3 + −	211.16 ± 22.27^a^	133.83 ± 7.22^a^	395.50 ± 2.16^a^	50.83 ± 2.83^a^

*Results are expressed as mean ± SEM, N = 6. Data were analyzed by one-way ANOVA, followed by Tukey (HSD). ^∗∗∗^p < 0.001, vs control animals (DW + DW group) receiving only distilled water; ^a^p < 0.05, ^b^p < 0.01, and ^c^p < 0.001 vs disease control animals (DW + Pilo group) receiving distilled water and pilocarpine (360 mg/kg). DW, distilled water; Cq, Cissus quadrangularis; Pilo, pilocarpine; SV, sodium valproate (300 mg/kg); DZP, diazepam (0.3 mg/kg).*

#### MDA Level

Malondiadéhyde level significantly increased from 129.83 ± 5.11 nmol/g tissue in the DW + DW group to 400.66 ± 4.44 nmol/g tissue in the DW + Pilo group. *C. quadrangularis* reversed the increase in MDA level from 400.66 ± 4.44 nmol/g in DW + Pilo group to 243.33 ± 4.44 (*p* < 0.001) and 141.16 ± 4.44 nmol/g of tissue (*p* < 0.001) at the respective doses of 186 and 372 mg/kg. Sodium valproate and diazepam also reversed the increase of MDA induced by pilocarpine (*p* < 0.001; **Table 3**).

#### GABA Level

The GABA level decreased from 397.50 ± 1.66 μg/g tissue of DW + DW to 257.16 ± 8.44 μg/g tissue (*p* < 0.001) in the DW + Pilo group. *C. quadrangularis* induced a dose-dependent increase in GABA level. The highest dose of *C. quadrangularis* induced an increase up to 393.66 + 5.77 μg/g tissue (*p* < 0.001) compared to the DW + Pilo group. The GABA level was also increased in sodium valproate and diazepam groups (*p* < 0.001), compared to the ED + Pilo group (**Table 3**).

#### GABA-T Activity Level

GABA-transaminase activity increased from 47.16 ± 2.11 pg/ min/mg tissue in ED + ED group to 112.33 ± 4.88 pg/min/mg tissue (*p* < 0.001) in ED group + Pilo. *C. quadrangularis* reversed the increased GABA-T activity from 112.33 ± 4.88 pg/min/mg tissue in the ED + Pilo group to 63.16 ± 2.16 and 45.83 ± 2.83 pg/min/mg tissue (*p* < 0.001) at the respective doses of 186 and 372 mg/kg of the extract. Sodium valproate and diazepam also reduced GABA-T activity 52.66 + 5.11 and 50.83 + 2.83 pg/min/mg tissue (*p* < 0.001) compared to the ED + pilo group (**Table 3**).

## Discussion

This study aimed to show antiepileptogenic and anticonvulsant effects of *C. quadrangularis* accompanied by its anxiolytic effects since anxiety is also developed in epileptic patients (Robertson et al., 1987; Torta and Keller, 1999; Mondon et al., 2002). Administration of pilocarpine, an experimental seizures inducer, induced seizures in mice according to the Racine scale (Racine, 1972). *C. quadrangularis* administered to mice at different doses though was not able to stop the development of seizures, but it delayed the onset of epileptic status induced by pilocarpine (Kandeda et al., 2017). One hour after its administration, *C. quadrangularis* delayed the onset of SE. Also 24 h after pilocarpine administration, *C. quadrangularis* increased the latency of the first clonic and tonic–clonic seizures, and decreased the number and duration of clonic and tonic–clonic seizures. These effects similar to the effects of sodium valproate (which is an antiepileptic reference substance) show that *C. quadrangularis* possesses anticonvulsant effects. This result is in accordance with the results of Ngo Bum et al. (2008), who showed that *C.* *quadrangularis* has anticonvulsant or antiepileptic properties by antagonizing pentylene tetrazol, strychnine, and picrotoxine induced convulsions and inhibiting NMDA-induced behavioral excitation (Schmutz et al., 1990; Meldrum, 1992; Bum et al., 2011). The antiepileptogenesis effect of *C. quadrangularis* was not clear since the induced epiloptogenesis was developed. Phytochemical studies of *C. quadrangularis* have revealed the presence of flavonoids (Shirley and Sen, 1966; Enechi and Odonwodo, 2003; Jainu et al., 2006), such as quercetins and kaempferols (Sen, 1966; Saburi et al., 1999; Amos et al., 2001), triterpenoids, and vitamin C. The presence of phenolic compounds and triterpenoids may partly explain the anticonvulsant activity of the plant as they are possessing actions against tonic–clonic seizures, respectively (Taviano et al., 2007).

In the EPM test, the number of entries, the percentages of entries, and time spent in the open arms increased in the presence of the extract, sodium valproate, and diazepam. In contrast, extract, valproate, and diazepam reduced the number of entries, the percentages of entries, and time spent in closed arms. An increase in the activity of mice in the open arms refers to a decrease in anxiety (Pitchaiah et al., 2008; Moto et al., 2013). And a decrease in these behavioral parameters in closed arms reflects a reduction in stress (Lister, 1990; Ngo Bum et al., 2009). This suggests that the aqueous extract of *C. quadrangularis* has anxiolytic properties. The decrease in the number of rearing and head dipping in the labyrinth, which indicates a decrease in anxiety (Rodgers et al., 1997; Ngo Bum et al., 2009), also contributes to the presence of anxiolytic effects of *C. quadrangularis*. Likewise, the effects of the extract are similar to those of diazepam (Löscher, 2012), which is a reference anxiolytic compound. The anxiolytic effects of *C. quadrangularis* would be mediated by GABAergic neurotransmission for EPM which is sensitive to benzodiazepines compounds.

The results obtained through the open arena test show that, like diazepam, the decoction increases the number of crossing, the number of grooming, and the time spent in the center of the device. The increase crossing, grooming, and time spent in the center of the open arena is a manifestation of the increase of locomotor and explorative activities in mice (Spruijt et al., 1992; Jenck et al., 1997; Kash et al., 1999; Augustsson, 2004; Ngo Bum et al., 2009; Moto et al., 2013). Because the total and closed arms entries and rearing in the EPM and rearing in OP were reduced (reduction of locomotion), the effect of the plant in this paradigm suggests mainly explorative activities which express the decrease in anxiety (File and Wardill, 1975; Jenck et al., 1997, Ngo Bum et al., 2009), so the decoction has anxiolytic properties. The significant decrease in stool production in mice treated with the decoction also suggests the presence of anxiolytic effects. Studies have shown that decreased stool mass is a result of lower stress (Rodgers et al., 1997) and any substance that reduces stress under stress conditions as imposed by the open arena is anxiolytic.

The aqueous extract of *C. quadrangularis* reduces the level of MDA and increases the level of GSH. Plasma MDA is a marker of lipid oxidation. It is known that MDA is one of the end products of the oxidation of polyunsaturated fatty acids. Therefore, a high level of MDA is an indicator of oxidative stress and cellular damage (Marnett, 1999). Similarly, a low or very high level of GSH is the manifestation of oxidative stress because GSH by reducing hydrogen peroxide prevents any deleterious effect such as lipid peroxidation (Lu, 2013). MDA and GSH are lipid peroxidation indices (lipid oxidation by ROS; Sivalokanathan et al., 2006). The drop in MDA and the increase in GSH reveal an antioxidant effect of the plant (Pryor and Stanley, 1975; Neve et al., 1989; Marnett, 1999; Sivalokanathan et al., 2006). Quercetin has been shown to have antioxidant activity. Quercetin decreases the production of ROS during moderate oxidative stress, improves the viability of H2O2-exposed neurons, and effectively inhibits cell apoptosis and hippocampal nerve cells (Suematsu et al., 2011; Jazvinscak Jembrek et al., 2012). Quercetin may partly explain the antioxidant effect of *C. quadrangularis*. The increase in GABA level and a decrease in GABA-T activity by the aqueous extract of *C. quadrangularis* were observed and suggest the interaction of the plant extract with the gabaergic neurotransmission, in the anticonvulsant effects of the plant. Sodium valproate induces the increase of cerebral GABA by the inhibition of semialdehyde succinyl dehydrogenase (a GABA degrading enzyme or GABA-T) or by the activation of GABA synthesis by glutamic acid decarboxylase (Hashimoto et al., 2006). This enzyme decreases the level of GABA in the brain and at the same time increases the level of the excitatory neurotransmitter glutamate, thereby causing the excitation of neurons (Johannessen, 2000). These results suggest that the extract would be able to restore and maintain the balance between neuronal excitation and inhibition and thus have anticonvulsant and anxiolytic activities.

## Conclusion

In this work, the effects of AECQ on pre- and post-SE seizures, anxiety disorders, and oxidative activity of mice in epileptogenesis were shown. AECQ significantly increased pre- and post-SE latency and decreased the number and duration of tonic–clonic seizures. The EACQ increased the number of entries and the time spent in the open arm of the EPM. Decreases in GSH and GABA levels and increases in MDA and GABA-T levels were normalized in AECQ-treated mice. AECQ has been shown to be an anticonvulsant extract with anxiolytic effects in animal models of epilepsy.

## Author Contributions

FM, JN, AA, and GT performed all behavioral studies, accomplished the data analysis, and drafted the manuscript. AK, ENB, SP, and GT designed the study. ENB critically revised the manuscript for important intellectual content. FM, GwN, GiN, JN, JOO, SP, and NK helped in *in vivo* studies. All authors read and approved the final manuscript.

## Conflict of Interest Statement

The authors declare that the research was conducted in the absence of any commercial or financial relationships that could be construed as a potential conflict of interest.
